# 1-(4-Chloro­phen­yl)-3-{5-[(*E*)-2-phenyl­ethen­yl]-1,3,4-thia­diazol-2-yl}urea

**DOI:** 10.1107/S1600536811002479

**Published:** 2011-01-29

**Authors:** Xiu-Huan Zhan, Zi-Yun Wang

**Affiliations:** a Department of Chemistry, Zhoukou Normal University, Zhoukou 466000, People’s Republic of China

## Abstract

In the title compound, C_17_H_13_ClN_4_OS, the 1,3,4-thia­diazole ring makes dihedral angles of 9.70 (15) and 7.22 (10)° with the benzene and phenyl rings, respectively; the dihedral angle between these two rings is 6.37 (19)°. In the crystal, pairs of N—H⋯N and C—H⋯O hydrogen bonds between inversion-related mol­ecules result in supra­molecular ribbons displaying alternate *R*_2_^2^(8) and *R*_2_^2^(14) graph-set ring motifs.

## Related literature

For the biological activity of urea derivatives, see: Abad *et al.* (2004 ▶); Chen *et al.* (2005 ▶); Yonova & Stoilkova (2005 ▶). For the biological activity of 1,3,4-thia­diazole derivatives, see: Guzeldemirci & Kucukbasmaci (2010 ▶); Song & Tan (2008 ▶); Zou *et al.* (2002 ▶). For the synthesis, see: Song *et al.* (2007 ▶).
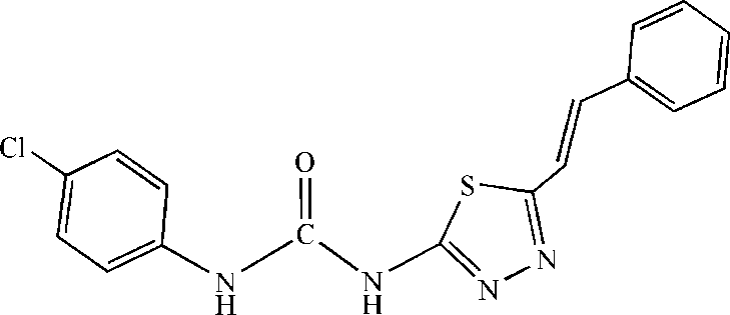


## Experimental

### 

#### Crystal data

C_17_H_13_ClN_4_OS
*M* *_r_* = 356.82
Monoclinic, 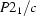

*a* = 11.2399 (6) Å
*b* = 4.1032 (2) Å
*c* = 35.2497 (16) Å
β = 91.525 (4)°
*V* = 1625.12 (14) Å^3^
*Z* = 4
Mo *K*α radiation
μ = 0.38 mm^−1^
*T* = 298 K
0.40 × 0.06 × 0.02 mm

#### Data collection

Bruker SMART CCD area-detector diffractometer
10860 measured reflections
3708 independent reflections
2081 reflections with *I* > 2σ(*I*)
*R*_int_ = 0.091

#### Refinement

*R*[*F* ^2^ > 2σ(*F* ^2^)] = 0.068
*wR*(*F* ^2^) = 0.154
*S* = 1.01
3708 reflections
223 parameters
H atoms treated by a mixture of independent and constrained refinement
Δρ_max_ = 0.31 e Å^−3^
Δρ_min_ = −0.20 e Å^−3^

### 

Data collection: *SMART* (Bruker, 2001 ▶); cell refinement: *SAINT* (Bruker, 2001 ▶); data reduction: *SAINT*; program(s) used to solve structure: *SHELXS97* (Sheldrick, 2008 ▶); program(s) used to refine structure: *SHELXL97* (Sheldrick, 2008 ▶); molecular graphics: *SHELXTL* (Sheldrick, 2008 ▶); software used to prepare material for publication: *SHELXTL*.

## Supplementary Material

Crystal structure: contains datablocks global, I. DOI: 10.1107/S1600536811002479/is2664sup1.cif

Structure factors: contains datablocks I. DOI: 10.1107/S1600536811002479/is2664Isup2.hkl

Additional supplementary materials:  crystallographic information; 3D view; checkCIF report

## Figures and Tables

**Table 1:** Hydrogen-bond geometry (Å, °)

*D*—H⋯*A*	*D*—H	H⋯*A*	*D*⋯*A*	*D*—H⋯*A*
C2—H2⋯O1^i^	0.93	2.55	3.432 (4)	159
N2—H2*A*⋯N3^ii^	0.82 (4)	2.03 (4)	2.848 (4)	174 (3)

Symmetry codes: (i) 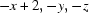; (ii) 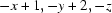.

**Fig. 1.**
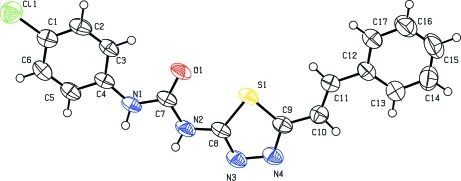
View of the title molecule, showing the atom-labelling scheme. Displacement ellipsoids are drawn at the 50% probability level. H atoms are represented by circles of arbitrary size.

**Fig. 2.**
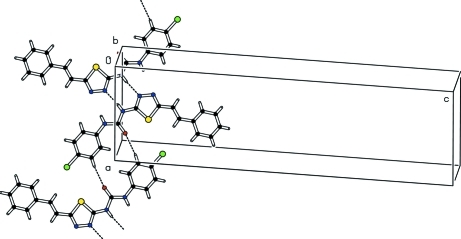
A partial packing diagram of the title molecule. Hydrogen bonds are indicated by dashed lines.

## supplementary crystallographic information

### Comment

Urea derivatives have attracted much attention on the account of their interesting biological effects, such as insecticidal, fungicidal, herbicidal and plant-growth regulating activities (Abad *et al.*, 2004; Chen *et al.*, 2005), especially cytokinin acyivity (Yonova & Stoilkova, 2005). 1,3,4-Thiadiazole derivatives are known to exhibit a wide range of biological activities (Zou *et al.*, 2002; Song & Tan, 2008; Guzeldemirci & Kucukbasmaci, 2010). In view of our extensive interest and as a continuing search for new urea-type cytokinins, we investigate the urea derivatives incorporating a 1,3,4-thiadiazole nucleus, including the title compound.

The crystal structure (Fig.1) revealed that the title molecule which consists of three rings is approximately planar, the dihedral angles formed by the thiadiazole ring with the chlorophenyl and vinylphenyl rings being only 9.70 (15) and 7.22 (10)°, respectively, and the styryl moiety assumes a *trans*-configuration about C10═C11 double bond of the vinyl moiety. All bond lengths and angles are as expected. In the crystal structure, intermolecular N—H···N and C—H···O hydrogen bonds occurring between centrosymmetrically related molecules result in the formation of ribbons displaying alternate rings of graph-set motifs *R*_2_^2^(8) and *R*_2_^2^(14), as shown in Fig. 2 and Table 1.

### Experimental

The title compound was prepared according to the procedure of Song *et al.* (2007). Suitable crystals were obtained by vapor diffusion of methanol in DMF at room temperature (m.p. >573 K). Elemental analysis: analysis calculated for C_17_H_13_ClN_4_OS: C 57.22, H 3.67, N 15.70%; found: C 57.45, H 3.56, N 15.82%.

### Refinement

C-bound H atoms were positioned geometrically and constrained to ride on their parent atoms, with C—H = 0.93 Å, and with *U*_iso_(H) = 1.2*U*_eq_(C). N-bound H atoms were freely refined [refined distances 0.82 (4) and 0.86 (3) Å].

### Crystal data

**Table:**

C_17_H_13_ClN_4_OS	*F*(000) = 736
*M**_r_* = 356.82	*D*_x_ = 1.458 Mg m^−^^3^*D*_m_ = 1.459 Mg m^−^^3^*D*_m_ measured by not measured
Monoclinic, *P*2_1_/*c*	Melting point > 573 K
Hall symbol: -P 2ybc	Mo *K*α radiation, λ = 0.71073 Å
*a* = 11.2399 (6) Å	Cell parameters from 1521 reflections
*b* = 4.1032 (2) Å	θ = 2.9–23.2°
*c* = 35.2497 (16) Å	µ = 0.38 mm^−^^1^
β = 91.525 (4)°	*T* = 298 K
*V* = 1625.12 (14) Å^3^	Block, colorless
*Z* = 4	0.40 × 0.06 × 0.02 mm

### Data collection

**Table:**

Bruker SMART CCD area-detector diffractometer	2081 reflections with *I* > 2σ(*I*)
Radiation source: fine-focus sealed tube	*R*_int_ = 0.091
graphite	θ_max_ = 27.5°, θ_min_ = 1.8°
φ and ω scans	*h* = −14→14
10860 measured reflections	*k* = −5→5
3708 independent reflections	*l* = −45→35

### Refinement

**Table:**

Refinement on *F*^2^	Primary atom site location: structure-invariant direct methods
Least-squares matrix: full	Secondary atom site location: difference Fourier map
*R*[*F*^2^ > 2σ(*F*^2^)] = 0.068	Hydrogen site location: inferred from neighbouring sites
*wR*(*F*^2^) = 0.154	H atoms treated by a mixture of independent and constrained refinement
*S* = 1.01	*w* = 1/[σ^2^(*F*_o_^2^) + (0.0498*P*)^2^] where *P* = (*F*_o_^2^ + 2*F*_c_^2^)/3
3708 reflections	(Δ/σ)_max_ = 0.001
223 parameters	Δρ_max_ = 0.31 e Å^−^^3^
0 restraints	Δρ_min_ = −0.20 e Å^−^^3^

### Special details

**Table:**

Geometry. All e.s.d.'s (except the e.s.d. in the dihedral angle between two l.s. planes) are estimated using the full covariance matrix. The cell e.s.d.'s are taken into account individually in the estimation of e.s.d.'s in distances, angles and torsion angles; correlations between e.s.d.'s in cell parameters are only used when they are defined by crystal symmetry. An approximate (isotropic) treatment of cell e.s.d.'s is used for estimating e.s.d.'s involving l.s. planes.
Refinement. Refinement of *F*^2^ against ALL reflections. The weighted *R*-factor *wR* and goodness of fit *S* are based on *F*^2^, conventional *R*-factors *R* are based on *F*, with *F* set to zero for negative *F*^2^. The threshold expression of *F*^2^ > σ(*F*^2^) is used only for calculating *R*-factors(gt) *etc*. and is not relevant to the choice of reflections for refinement. *R*-factors based on *F*^2^ are statistically about twice as large as those based on *F*, and *R*- factors based on ALL data will be even larger.

### Fractional atomic coordinates and isotropic or equivalent isotropic displacement parameters (Å^2^)

**Table:**

	*x*	*y*	*z*	*U*_iso_*/*U*_eq_	
C1	0.9546 (3)	−0.0487 (8)	−0.10673 (10)	0.0517 (9)	
C2	0.9869 (3)	0.0032 (9)	−0.06905 (10)	0.0559 (10)	
H2	1.0585	−0.0780	−0.0592	0.067*	
C3	0.9118 (3)	0.1766 (8)	−0.04629 (10)	0.0519 (9)	
H3	0.9330	0.2130	−0.0210	0.062*	
C4	0.8049 (3)	0.2972 (8)	−0.06083 (10)	0.0440 (8)	
C5	0.7739 (3)	0.2341 (9)	−0.09865 (10)	0.0536 (9)	
H5	0.7012	0.3078	−0.1085	0.064*	
C6	0.8486 (3)	0.0657 (9)	−0.12150 (10)	0.0565 (10)	
H6	0.8277	0.0291	−0.1468	0.068*	
C7	0.7271 (3)	0.5485 (8)	−0.00213 (10)	0.0468 (8)	
C8	0.6130 (3)	0.8377 (8)	0.04418 (10)	0.0458 (8)	
C9	0.5972 (3)	0.9536 (8)	0.10951 (9)	0.0470 (8)	
C10	0.6089 (3)	0.9901 (8)	0.15047 (10)	0.0499 (9)	
H10	0.5513	1.1121	0.1626	0.060*	
C11	0.6956 (3)	0.8619 (8)	0.17178 (10)	0.0491 (9)	
H11	0.7501	0.7342	0.1591	0.059*	
C12	0.7170 (3)	0.8949 (8)	0.21285 (10)	0.0505 (9)	
C13	0.6371 (4)	1.0434 (9)	0.23675 (11)	0.0639 (10)	
H13	0.5660	1.1267	0.2268	0.077*	
C14	0.6625 (5)	1.0679 (11)	0.27489 (12)	0.0839 (13)	
H14	0.6079	1.1654	0.2907	0.101*	
C15	0.7677 (5)	0.9501 (11)	0.28999 (12)	0.0881 (15)	
H15	0.7843	0.9683	0.3159	0.106*	
C16	0.8480 (4)	0.8058 (11)	0.26689 (13)	0.0810 (13)	
H16	0.9197	0.7273	0.2770	0.097*	
C17	0.8222 (4)	0.7772 (9)	0.22848 (11)	0.0656 (11)	
H17	0.8768	0.6767	0.2129	0.079*	
Cl1	1.05145 (9)	−0.2566 (3)	−0.13606 (3)	0.0742 (4)	
N1	0.7246 (2)	0.4851 (7)	−0.04021 (8)	0.0500 (8)	
H1A	0.670 (3)	0.583 (8)	−0.0530 (9)	0.060*	
N2	0.6343 (3)	0.7450 (8)	0.00788 (9)	0.0540 (8)	
H2A	0.588 (3)	0.822 (8)	−0.0081 (10)	0.065*	
N3	0.5194 (2)	1.0175 (7)	0.05141 (8)	0.0548 (8)	
N4	0.5103 (2)	1.0847 (7)	0.08984 (8)	0.0539 (8)	
O1	0.7993 (2)	0.4425 (6)	0.02092 (6)	0.0620 (7)	
S1	0.69949 (7)	0.7370 (2)	0.08328 (2)	0.0497 (3)	

### Atomic displacement parameters (Å^2^)

**Table:**

	*U*^11^	*U*^22^	*U*^33^	*U*^12^	*U*^13^	*U*^23^
C1	0.045 (2)	0.051 (2)	0.060 (2)	−0.0060 (17)	−0.0008 (18)	0.0119 (18)
C2	0.0382 (19)	0.064 (2)	0.065 (3)	0.0002 (18)	−0.0084 (18)	0.019 (2)
C3	0.042 (2)	0.065 (2)	0.047 (2)	−0.0022 (17)	−0.0103 (16)	0.0139 (18)
C4	0.0360 (17)	0.046 (2)	0.050 (2)	−0.0055 (15)	−0.0075 (15)	0.0151 (17)
C5	0.043 (2)	0.064 (2)	0.052 (2)	−0.0041 (18)	−0.0138 (17)	0.012 (2)
C6	0.057 (2)	0.065 (2)	0.047 (2)	−0.0064 (19)	−0.0108 (19)	0.0081 (19)
C7	0.0388 (19)	0.051 (2)	0.050 (2)	−0.0031 (16)	−0.0126 (16)	0.0128 (18)
C8	0.0393 (19)	0.050 (2)	0.048 (2)	−0.0026 (15)	−0.0126 (15)	0.0128 (17)
C9	0.0367 (18)	0.052 (2)	0.052 (2)	−0.0047 (15)	−0.0042 (16)	0.0126 (17)
C10	0.0432 (19)	0.054 (2)	0.053 (2)	−0.0010 (17)	0.0034 (17)	0.0024 (18)
C11	0.048 (2)	0.049 (2)	0.051 (2)	−0.0004 (16)	−0.0034 (17)	0.0044 (17)
C12	0.057 (2)	0.050 (2)	0.044 (2)	−0.0060 (17)	−0.0064 (18)	0.0063 (17)
C13	0.071 (3)	0.068 (3)	0.053 (3)	0.007 (2)	−0.001 (2)	0.001 (2)
C14	0.120 (4)	0.074 (3)	0.059 (3)	0.007 (3)	0.012 (3)	−0.009 (2)
C15	0.142 (5)	0.071 (3)	0.051 (3)	−0.006 (3)	−0.023 (3)	−0.001 (2)
C16	0.099 (4)	0.076 (3)	0.066 (3)	0.003 (3)	−0.025 (3)	0.003 (3)
C17	0.070 (3)	0.072 (3)	0.054 (2)	0.007 (2)	−0.011 (2)	0.004 (2)
Cl1	0.0655 (7)	0.0789 (7)	0.0785 (8)	0.0047 (5)	0.0083 (5)	0.0018 (6)
N1	0.0393 (16)	0.060 (2)	0.0493 (19)	0.0041 (14)	−0.0180 (14)	0.0090 (15)
N2	0.0420 (17)	0.070 (2)	0.049 (2)	0.0067 (15)	−0.0162 (13)	0.0101 (17)
N3	0.0385 (16)	0.071 (2)	0.0543 (19)	0.0026 (15)	−0.0145 (14)	0.0089 (16)
N4	0.0385 (16)	0.066 (2)	0.057 (2)	−0.0004 (14)	−0.0077 (14)	0.0098 (16)
O1	0.0511 (15)	0.0825 (18)	0.0513 (15)	0.0170 (13)	−0.0186 (12)	0.0107 (14)
S1	0.0397 (5)	0.0609 (6)	0.0479 (5)	0.0024 (4)	−0.0117 (4)	0.0111 (5)

### Geometric parameters (Å, °)

**Table:**

C1—C6	1.370 (4)	C9—S1	1.739 (3)
C1—C2	1.384 (5)	C10—C11	1.324 (4)
C1—Cl1	1.744 (4)	C10—H10	0.9300
C2—C3	1.378 (5)	C11—C12	1.468 (4)
C2—H2	0.9300	C11—H11	0.9300
C3—C4	1.385 (4)	C12—C17	1.379 (5)
C3—H3	0.9300	C12—C13	1.388 (5)
C4—C5	1.393 (5)	C13—C14	1.370 (5)
C4—N1	1.404 (4)	C13—H13	0.9300
C5—C6	1.366 (5)	C14—C15	1.372 (6)
C5—H5	0.9300	C14—H14	0.9300
C6—H6	0.9300	C15—C16	1.366 (6)
C7—O1	1.214 (4)	C15—H15	0.9300
C7—N1	1.367 (4)	C16—C17	1.382 (5)
C7—N2	1.372 (4)	C16—H16	0.9300
C8—N3	1.316 (4)	C17—H17	0.9300
C8—N2	1.362 (4)	N1—H1A	0.86 (3)
C8—S1	1.716 (3)	N2—H2A	0.82 (4)
C9—N4	1.299 (4)	N3—N4	1.389 (4)
C9—C10	1.454 (4)		
			
C6—C1—C2	121.0 (3)	C10—C11—C12	128.5 (3)
C6—C1—Cl1	119.5 (3)	C10—C11—H11	115.8
C2—C1—Cl1	119.5 (3)	C12—C11—H11	115.8
C3—C2—C1	119.3 (3)	C17—C12—C13	118.2 (3)
C3—C2—H2	120.4	C17—C12—C11	118.6 (3)
C1—C2—H2	120.4	C13—C12—C11	123.2 (3)
C2—C3—C4	120.5 (3)	C14—C13—C12	120.4 (4)
C2—C3—H3	119.7	C14—C13—H13	119.8
C4—C3—H3	119.7	C12—C13—H13	119.8
C3—C4—C5	118.7 (3)	C13—C14—C15	120.6 (4)
C3—C4—N1	124.6 (3)	C13—C14—H14	119.7
C5—C4—N1	116.8 (3)	C15—C14—H14	119.7
C6—C5—C4	121.1 (3)	C16—C15—C14	119.8 (4)
C6—C5—H5	119.4	C16—C15—H15	120.1
C4—C5—H5	119.4	C14—C15—H15	120.1
C5—C6—C1	119.4 (3)	C15—C16—C17	119.7 (4)
C5—C6—H6	120.3	C15—C16—H16	120.1
C1—C6—H6	120.3	C17—C16—H16	120.1
O1—C7—N1	125.8 (3)	C12—C17—C16	121.1 (4)
O1—C7—N2	122.6 (3)	C12—C17—H17	119.4
N1—C7—N2	111.6 (3)	C16—C17—H17	119.4
N3—C8—N2	120.0 (3)	C7—N1—C4	128.1 (3)
N3—C8—S1	114.7 (3)	C7—N1—H1A	115 (2)
N2—C8—S1	125.3 (3)	C4—N1—H1A	117 (2)
N4—C9—C10	122.3 (3)	C8—N2—C7	124.0 (3)
N4—C9—S1	115.2 (3)	C8—N2—H2A	114 (2)
C10—C9—S1	122.5 (2)	C7—N2—H2A	122 (2)
C11—C10—C9	124.6 (3)	C8—N3—N4	112.3 (3)
C11—C10—H10	117.7	C9—N4—N3	111.4 (3)
C9—C10—H10	117.7	C8—S1—C9	86.31 (17)
			
C6—C1—C2—C3	−0.9 (5)	C13—C12—C17—C16	−0.1 (6)
Cl1—C1—C2—C3	177.9 (2)	C11—C12—C17—C16	179.0 (3)
C1—C2—C3—C4	0.2 (5)	C15—C16—C17—C12	0.7 (6)
C2—C3—C4—C5	1.2 (5)	O1—C7—N1—C4	−2.2 (6)
C2—C3—C4—N1	−177.8 (3)	N2—C7—N1—C4	179.4 (3)
C3—C4—C5—C6	−2.0 (5)	C3—C4—N1—C7	−9.2 (5)
N1—C4—C5—C6	177.1 (3)	C5—C4—N1—C7	171.7 (3)
C4—C5—C6—C1	1.4 (5)	N3—C8—N2—C7	−177.6 (3)
C2—C1—C6—C5	0.1 (5)	S1—C8—N2—C7	2.0 (5)
Cl1—C1—C6—C5	−178.7 (3)	O1—C7—N2—C8	−0.7 (5)
N4—C9—C10—C11	−179.9 (3)	N1—C7—N2—C8	177.8 (3)
S1—C9—C10—C11	−0.9 (5)	N2—C8—N3—N4	179.0 (3)
C9—C10—C11—C12	177.6 (3)	S1—C8—N3—N4	−0.6 (4)
C10—C11—C12—C17	−171.1 (3)	C10—C9—N4—N3	179.5 (3)
C10—C11—C12—C13	7.9 (6)	S1—C9—N4—N3	0.4 (4)
C17—C12—C13—C14	−0.7 (6)	C8—N3—N4—C9	0.1 (4)
C11—C12—C13—C14	−179.7 (4)	N3—C8—S1—C9	0.7 (3)
C12—C13—C14—C15	0.8 (7)	N2—C8—S1—C9	−178.9 (3)
C13—C14—C15—C16	−0.2 (7)	N4—C9—S1—C8	−0.6 (3)
C14—C15—C16—C17	−0.5 (7)	C10—C9—S1—C8	−179.7 (3)

### Hydrogen-bond geometry (Å, °)

**Table:**

*D*—H···*A*	*D*—H	H···*A*	*D*···*A*	*D*—H···*A*
C2—H2···O1^i^	0.93	2.55	3.432 (4)	159
N2—H2A···N3^ii^	0.82 (4)	2.03 (4)	2.848 (4)	174 (3)

Symmetry codes: (i) −*x*+2, −*y*, −*z*; (ii) −*x*+1, −*y*+2, −*z*.
